# Increasing *Candida* antifungal resistance in Eastern India (2019-2023): A notable rise in amphotericin B resistance

**DOI:** 10.22034/cmm.2024.345262.1555

**Published:** 2024-11-15

**Authors:** Poulami Mukherjee, Purushottam Dutta, Apoorbaa Roy, Prabuddha Mukhopadhyay

**Affiliations:** 1 Department of Microbiology, Ramakrishna Mission Seva Pratisthan/Vivekananda Institute of Medical Sciences, Kolkata, West Bengal, India; 2 Department of General Medicine, Ramakrishna Mission Seva Pratisthan/Vivekananda Institute of Medical Sciences, Kolkata, West Bengal, India

**Keywords:** Antifungal susceptibility, Amphotericin B, *Candida*, Non-*albicans Candida* species

## Abstract

**Background and Purpose:**

This study analyzed the species diversity and antifungal drug sensitivity of *Candida* isolates reported from 2019 to 2023 at our hospital to guide empirical treatment protocols.

**Materials and Methods:**

Clinical samples were cultured by standard microbiological techniques; subsequently, yeast isolates deemed clinically significant were identified and tested for antifungal drug sensitivity using the Vitek 2 Compact system (BioMerieux, France). Statistical analysis was performed in SPSS software and *P*-values of less than 0.05 were considered statistically significant.

**Results:**

Diversity of species as well as the antifungal drug resistance of *Candida* isolates increased markedly over the period of 5 years. Moreover, a high percentage of isolates resistant to Amphotericin B and Voriconazole was noted.

**Conclusion:**

These findings emphasized the need for caution in the empirical use of antifungal medications. Similar surveillance at regional levels is necessary and antifungal drug sensitivity should be included in hospital antibiograms to prevent the spread of multi-drug-resistant nosocomial strains.

## Introduction

*Candida* species are frequent opportunistic and nosocomial pathogens in immunocompromised patients [ 1 ], associated with high mortality rates and prolonged hospital stay [ 2 ]. Worldwide studies have reported non-*albicans Candida* (NAC) species, such as *C. glabrata*, *C. tropicalis*, *C. guilliermondii*, *C. krusei*, *C. lusitaniae*, and *C. parapsilosis* as emerging pathogens, which are resistant to multiple antifungal agents [ 3 - 6 ]. Moreover, *C. krusei* and *C. glabrata* have shown reduced susceptibility to fluconazole [ 8 ] while *C. lusitaniae* are often resistant to amphotericin B [ 7 ], and intrinsically reduced susceptibility to echinocandins is seen in *C. parapsilosis* and *C. guilliermondii* [ 8 ]. Based on previous research, *C. auris*, a multidrug-resistant, healthcare-associated pathogen with a high mortality rate has particularly been a cause of concern [ 9 ]. Prolonged use of antifungal medications for prophylaxis or treatment of recurrent fungal infections in immunocompromised patients is a major factor leading to the emergence of antifungal resistance [ 10 ]. The epidemiological changes in the prevalence of the different *Candida* species causing systemic infections have been in parallel to the emergence of drug-resistant species [ 11 ]. In patients who received antifungal prophylaxis, NAC species were more commonly isolated than *C. albicans* [ 12 ]. During the COVID-19 pandemic, there was an increase in invasive fungal infections and subsequently the use of antifungal agents. Therefore, there have been reports of an increase in resistance to antifungal medications in the years following the pandemic [ 13 ]. This has led to a pressing need for continuous surveillance at the regional level to guide treatment. With this aim, the present study retrospectively analyzed the species distribution of *Candida* isolates and the pattern of antifungal drug susceptibility from various clinical samples at an urban tertiary care hospital in Eastern India, over a period of 5 years (2019- 2023).

## Materials and Methods

This study received ethical approval from the institutional Ethical Committee (Registration number: ECR/62/Inst/WB/2013/RR-19). Samples from in-patient and out-patient departments (IPD and OPD) of our hospital in eastern India were processed in the hospital laboratory. Urine, high vaginal swabs, and blood cultures constituted the majority of the samples. Respiratory samples were not included in the study as *Candida* species are common colonizers of the respiratory tract and their role as pathogens is controversial [ 14 ]. *Candida* in urine was only reported in clinically symptomatic cases with pyuria, yielding pure growth of *Candida* in cultures and confirmed by repeated cultures whenever possible. High vaginal swabs were received mainly from the Department of Obstetrics for symptomatic cases and any growth of *Candida* spp. in these samples was reported since vaginal candidiasis in pregnant women is a risk factor for bloodstream infections, particularly in low birth weight and premature infants. Any growth in blood cultures and other samples was clinically correlated with the patient history. All *Candida* spp. that were reported over the period of 5 years (Jan 1, 2019, to December 31, 2023) were included in the study.

Samples were cultured on Sabouraud dextrose agar (SDA, HiMedia, India). Blood samples were incubated in blood culture bottles by BactAlert3D (Biomerieux, France) automated culture system. Bottles giving positive alarm within 5 days were subcultured on SDA and blood agar plates (Biomeriuex, France). Any yeast isolates were identified using the Vitek2 Compact system (Biomerieux, France) via ID-YST panel for identification and AST-YS08 panel for antifungal susceptibility testing to Amphotericin B, Caspofungin, Flucytosine, Fluconazole, Micafungin, and Voriconazole. The Clinical and Laboratory Standards Institute (CLSI M60, 2^nd^ Edition, June 2020) and European Committee on Antimicrobial Susceptibility Testing (EUCAST) (Version 10.0, February 2020) breakpoints were used for the interpretation of the Vitek minimum inhibitory concentration (MIC) data. For the *C. auris* isolates, MICs were determined by broth microdilution method and interpreted using Centre for Disease Control (CDC) breakpoints as CLSI and EUCAST breakpoints were not defined. Statistical analysis was performed in SPSS software. Pearson's chi-squared test, Kruskal Wallis test, and one-way ANOVA tests were used to determine the association between categorical variables. It should be mentioned that P-values of less than 0.05 were considered statistically significant.

## Results and Discussion

In total, 520 *Candida* species cultured from clinical samples were included in this study. Types of samples from which *Candida* was grown consisted of urine (68%) followed by high vaginal swabs (23.5%), blood (7.5%), and others (pus, ear swab, nasal mass biopsy tissue, and nappy rash) (1%). Majority of the samples were collected from the IPD (56%), followed by the intensive care units (ICU), high dependency units (HDU) (35%), and OPDs (9%). Based on findings, 24 (61.5%) out of 39 blood culture isolates were from the pediatric age group (<12 years) and 30 (77%) were from patients in critical care units (ICU, neonatal ICU, and Pediatric ICU).

**Figure 1:**
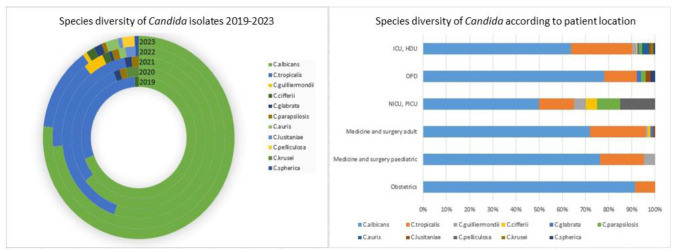
Species diversity of *Candida* isolated from clinical samples (2019-2023)

*Candida albicans* isolates vastly outnumbered all other species of *Candida* isolated (70%) followed by *C. tropicalis* (22.5%). Other isolated species were *C. guilliermondii*, *C. ciferrii*, *C. parapsilosis*, *C. glabrata*, *C. auris*, *C. lusitaniae* (~1% each), *C. pelliculosa*, *C. spherica*, and *C. krusei* (~0.5% each). Number of patients with *Candida* growth were 87, 61, 65,141, and 128 in 2019, 2020, 2021, 2022, and 2023, respectively. In 2019, only 3 species of *Candida* were identified from our hospital (*C. albicans*, *C. tropicalis*, and *C. ciferrii*) but the diversity increased over the years to 10 species in 2023 (with the addition of *C. auris*, *C. ciferrii*, *C. guilliermondii*, *C. glabrata*, *C. lusitaniae*, *C. spherica*, and *C. pelliculosa*) [Figure 1]. 

There were no *C. auris* isolated from 2019 to 2021, but between 2022 and 2023 there were five isolates of *C. auris*, all from patients in critical care units. One patient had *C. auris* isolated from both blood and urine denoting the likelihood of septicemia. However, the prevalence of *C. auris* in our center was much lower, compared to those reported in previous studies from India [ 15 - 17 ]. This could probably be due to the fact that these studies only considered samples from ICU setups. In terms of patient location, the least diverse samples were those collected from the obstetrics departments with only *C. albicans* and *C. tropicalis* being isolated while the most diverse samples, with a total of 10 species, were collected from the critical care departments (ICU and HDU) [Figure 1]. During the COVID-19 pandemic, patient intake and footfall were markedly reduced at the hospital due to unavailability of transportation, postponement of planned surgeries, and referral of COVID-19-positive cases to infectious disease facilities. However, it is evident that the number of cases of *Candida* infections remained quite high, and in the post-COVID period there was an increase in the number of cases which corroborated with reports of an increase in fungal infections as an aftermath of the COVID-19 pandemic [ 13 ].

Antifungal sensitivity patterns for *C. albicans* (365 isolates) and *C. tropicalis* (117 isolates) are summarized in Table 1.

**Table 1:** Percentage of resistant isolates of *Candida albicans* and *Candida tropicalis*

Name of drug	*Candida albicans*	*Candida tropicalis*	*P*- value	*P*-value	*Candida albicans*						*Candida tropicalis*					
	Total (5 years)	Total (5 years)			2019	2020	2021	2022	2023	*P* value	2019	2020	2021	2022	2023	*P* value
AMB	16.71	10.26	0.090	0.281	1.64	16.67	2.70	23.28	22.94	0.0002	3.85	5.26	7.14	20	15.79	0.275
CAS	7.40	8.55	0.684		1.64	2.38	0	12.93	9.17	0.010	0	5.26	14.29	12.0	10.53	0.369
5FC	5.71	8.94	0.056		0	0	0	10.34	4.59	0.003	0	10.34	8.33	12.0	15.79	0.306
FLC	5.21	6.84	0.504		0	2.38	2.70	11.21	3.67	0.008	0	15.79	7.14	8.00	5.26	0.359
MFG	7.40	9.40	0.483		3.28	2.38	0	12.07	9.17	0.036	7.69	0	14.29	12.0	10.53	0.556
VRC	13.70	5.13	0.011		6.56	11.9	8.11	17.24	16.51	0.225	0	5.26	7.14	8.00	5.26	0.733

AMB; Amphotericin B, CAS; Caspofungin, 5FC; Flucytosine, FLC; Fluconazole, MFG; Micafungin, Voriconazole; VRC

The other species were not included in this table due to their small numbers and the unavailability of complete CLSI and EUCAST breakpoints for many of them.

For *C. albicans*, the highest percentage of drug resistance over 5 years was observed against amphotericin B (17%) and voriconazole (14%), and in the case of *C. tropicalis* against amphotericin B (10%), caspofungin, flucytosine and micafungin (~9% each).

The MIC values for amphotericin B were also higher than those for the other antifungal agents. These findings differed from those of previous studies which found overall good sensitivity of *Candida* species to amphotericin B, and high resistance to the azoles, including fluconazole [ 18 - 25 ]. However, according to the CDC website, fluconazole resistance in *Candida* stands at around 7% which is similar to that observed in this study. There was no significant difference between *C. albicans* and *C. tropicalis* in terms of overall antifungal drug resistance (*P*=0.2819); however, when considering individual antifungal agents, *C. albicans* was significantly more resistant against voriconazole only, compared to *C. tropicalis* (*P*=0.0118). These findings are also in contrast to those of previous studies which described higher antifungal drug resistance in the NAC species, compared to *C. albicans*. 

For *C. albicans*, the percentage of resistant isolates increased significantly over the period of 5 years (*P*<0.05) for all agents except voriconazole. For *C. tropicalis*, the increase in the percentage of resistant organisms was not statistically significant for any of the antifungal agents [Table 1].

Organisms with resistance to three or more antifungal drugs were considered multi-drug-resistant organisms (MDROs), and their total number was 31. Number of MDROs progressively increased over 5 years: there were no MDROs in 2019, 2 in 2020, 3 in 2021, 17 in 2022, and 9 in 2023. Majority of the MDROs were *C. albicans* (23), and the rest were *C. tropicalis* (8). Amongst the MDROs, the lowest resistance was observed against fluconazole with 12 of the 31 MDROs being susceptible to it. 

Table 2 tabulates the MIC mode, MIC_50_, and MIC_90_ values of *C. albicans* and *C. tropicalis* each year. No significant change was noted in the mode of MIC values; however, there was a progressive increase in the MIC_90_ values, most markedly for amphotericin B for both organisms. 

**Table 2:** Mode, minimum inhibitory concentration 50% (MIC_50_), and MIC_90_ values for *C. albicans* and *C. tropicalis*

Antifungal agent	Organism	MIC range (ug/ml)	Overall			2019			2020			2021			2022			2023		
			Mode	MIC_50_	MIC_90_	Mode	MIC_50_	MIC_90_	Mode	MIC_50_	MIC_90_	Mode	MIC_50_	MIC_90_	Mode	MIC_50_	MIC_90_	Mode	MIC_50_	MIC_90_
AMB	*C. albicans*	≤0.25 - ≥16	1	1	4	1	1	1	1	1	4	1	1	1	1	1	8	1	1	8
	*C. tropicalis*	≤0.25 - ≥16	0.5	0.5	2	≤0.25	0.375	0.5	≤0.25	≤0.25	1	≤0.25	0.5	1	≤0.25	0.5	8	≤0.25	0.5	≥16
CAS	*C. albicans*	≤0.12 - ≥8	≤0.12	≤0.12	0.25	≤0.12	≤0.12	≤0.12	≤0.12	≤0.12	≤0.12	≤0.12	≤0.12	≤0.12	≤0.12	≤0.12	≥8	≤0.12	≤0.12	0.25
	*C. tropicalis*	≤0.12 - ≥8	≤0.12	≤0.12	0.5	≤0.12	≤0.12	0.25	≤0.12	≤0.12	0.25	≤0.12	≤0.12	≥8	≤0.12	≤0.12	≥8	≤0.12	≤0.12	≥8
5FC	*C. albicans*	≤1 - ≥64	≤1	≤1	≤1	≤1	≤1	≤1	≤1	≤1	≤1	≤1	≤1	≤1	≤1	≤1	≥64	≤1	≤1	≤1
	*C. tropicalis*	≤1 - ≥64	≤1	≤1	16	≤1	≤1	≤1	≤1	≤1	≥64	≤1	≤1	16	≤1	≤1	≥64	≤1	≤0.5	≥64
FLC	*C. albicans*	≤0.5 - ≥64	≤0.5	≤0.5	4	≤0.5	≤0.5	1	≤0.5	≤0.5	1	≤0.5	≤0.5	1	≤0.5	≤0.5	8	≤0.5	≤0.5	4
	*C. tropicalis*	≤0.5 - ≥64	≤0.5	1	2	≤0.5	≤0.5	2	1	1	16	1	1	4	≤0.5	≤0.5	2	≤0.5	≤0.5	2
MFG	*C. albicans*	≤0.06 - ≥8	≤0.06	≤0.06	≤0.06	≤0.06	≤0.06	≤0.06	≤0.06	≤0.06	≤0.06	≤0.06	≤0.06	≤0.06	≤0.06	≤0.06	≥8	≤0.06	≤0.06	0.12
	*C. tropicalis*	≤0.06 - ≥8	≤0.06	≤0.06	0.5	≤0.06	≤0.06	0.5	≤0.06	≤0.06	≤0.06	≤0.06	≤0.06	4	≤0.06	≤0.06	≥8	≤0.06	≤0.06	≥8
VRC	*C. albicans*	≤0.12 - ≥8	≤0.12	≤0.12	1	≤0.12	≤0.12	≤0.12	≤0.12	≤0.12	1	≤0.12	≤0.12	≤0.12	≤0.12	≤0.12	2	≤0.12	≤0.12	1
	*C. tropicalis*	≤0.12 - ≥8	≤0.12	≤0.12	≤0.12	≤0.12	≤0.12	≤0.12	≤0.12	≤0.12	≤0.12	≤0.12	≤0.12	≤0.12	≤0.12	≤0.12	≤0.12	≤0.12	≤0.12	≤0.12

AMB; Amphotericin B, CAS; Caspofungin, 5FC; Flucytosine, FLC; Fluconazole, MFG; Micafungin, Voriconazole; VRC

Out of the five isolates of *C. auris*, all were susceptible to fluconazole, caspofungin, and micafungin, while two isolates were resistant to amphotericin B. The CDC breakpoints for *C. auris* against flucytosine and voriconazole were not available.

Therefore, an overall increasing trend of antifungal drug resistance as well as increasing diversity of species was noted in this 5-year period.

## Conclusion

While antimicrobial drug resistance in bacteria is a major problem that is being addressed globally, fungal resistance to antifungal drugs is rarely highlighted and studied. In developing countries, such as India, empirical use of antifungal agents is common and has only increased following the rise of fungal infections during the COVID-19 pandemic. In this study, a considerable increase was found in the diversity of isolated species as well as the antifungal resistance over the period of 5 years in our hospital. Therefore, we should exercise caution in the use of antifungal drugs. Moreover, culture and sensitivity testing with robust clinical criteria for reporting fungal isolates should be followed in all suspected cases. Based on the findings of the present study, continuous surveillance of the prevalent species of *Candida* and their antifungal resistance patterns should be carried out in hospitals regularly, to guide treatment and prevent the spread of multidrug-resistant and nosocomial strains. A limitation of this study was its retrospective design which prevented the study of the mechanisms of drug resistance in these fungi, which is necessary to identify the means to overcome such resistance.
